# Exploring the intangible economic costs of stillbirth

**DOI:** 10.1186/s12884-015-0617-x

**Published:** 2015-09-01

**Authors:** Chidubem B. Ogwulu, Louise J. Jackson, Alexander E.P. Heazell, Tracy E. Roberts

**Affiliations:** Health Economics Unit, School of Health and Population Sciences, University of Birmingham, Edgbaston, Birmingham, B15 2TT UK; Institute of Human Development, Faculty of Medical and Human Sciences, University of Manchester and Manchester Academic Health Science Centre, St Mary’s Hospital, Oxford Road, Manchester, M13 9WL UK

## Abstract

**Background:**

Compared to other pregnancy-related events, the full cost of stillbirth remains poorly described. In the UK one in every 200 births ends in stillbirth. As a follow-up to a recent study which explored the direct costs of stillbirth, this study aimed to explore the intangible costs of stillbirth in terms of their duration and economic implication.

**Methods:**

Systematic searches identified relevant papers on the psychological consequences of stillbirth. A narrative review of the quantitative studies was undertaken. This was followed by a qualitative synthesis using meta-ethnography to identify over-arching themes common to the papers. Finally, the themes were used to generate questions proposed for use in a questionnaire to capture the intangible costs of stillbirth.

**Results:**

The narrative review revealed a higher level of anxiety and depression in couples with stillbirth compared to those without stillbirth. The qualitative synthesis identified a range of psychological effects common to families that have experienced stillbirth. Both methods revealed the persistent nature of these effects and the subsequent economic burden.

**Conclusions:**

The psychological effects of stillbirth adversely impacts on the daily functioning, relationships and employment of those affected with far-reaching economic implications. Knowledge of the intangible costs of stillbirth is therefore important to accurately estimate the size of the impact on families and health services and to inform policy and decision making.

**Electronic supplementary material:**

The online version of this article (doi:10.1186/s12884-015-0617-x) contains supplementary material, which is available to authorized users.

## Background

The World Health Organisation (WHO) defines stillbirth as “the birth of a baby with no signs of life at, or after 28 completed weeks of pregnancy” [1]. Globally, in 2009, around 3 million babies were stillborn [1] and in 2012, there were 3558 stillbirths in England and Wales [2]. The United Kingdom (UK) rate; defined as “the birth of a baby that has died after at least 24 completed weeks of pregnancy” is approximately five in every 1000 births [3], one of the highest in Europe and with little significant reduction in decades [4].

Stillbirth is a traumatic experience with reports of adverse psychosocial effects such as: anxiety, depression, shame, suicidal thoughts, post-traumatic stress disorder (PTSD) and guilt [5–7]. The overwhelming impact on parents can be long-lasting [8, 9] and ripples outwards to siblings, grandparents, extended family and friends [10]. In the long-term, it affects couples’ relationships, siblings, subsequent children, social life, career and work colleagues [11]. Thus it can further impact on the healthcare resources utilization of affected individuals [12].

However, stillbirth has been termed an ‘invisible death’ due to being neglected as a public health issue of importance to society and health policy makers [13]. Efforts to highlight its international importance include the publication of the Lancet Stillbirth Series [14]. Nevertheless, when compared to other pregnancy-related issues such as miscarriages, live-births and neonatal deaths, the consequences of stillbirth have not been well reported [15]. Therefore, its societal impact and relevance to health policy is underestimated and measures for its reduction are given little priority by policy makers [15].

The economic impact of an illness involves all costs and outcomes associated with its incidence. These include: direct costs - the monetary cost of all resources associated with the provision of an intervention e.g. health service use and medications [16], indirect costs - the value of output, lost productivity or forgone manpower resources incurred from time off work due to morbidity or disability following an illness [17], and intangible costs - non-monetary costs [18] reflecting the ‘disvalue’ to an individual of pain, anxiety, fear and suffering [19].

Direct and indirect costs do not attempt to reflect the range of deterioration in quality of life from interventions/diseases. However, the term intangible costs encompasses the psychological dimensions of illness [20] but they are difficult to quantify or to account for explicitly in economic models [21, 22]. In some circumstances, intangible costs might exceed the direct/indirect costs due to their impact on individual and societal welfare [18]. Therefore intangible costs potentially play a major role in patients’ healthcare decisions and in decision making for resource allocation [23].

A recent paper by Mistry et al. [24] classified the economic costs to the health service due to stillbirths into three groups: i) the direct costs of immediate care after stillbirth; ii) costs incurred after the completion of initial management; iii) costs incurred in a subsequent pregnancy. Despite using a quantitative and systematic approach, the authors found direct evidence only for groups (i) and (iii). They identified the costs incurred in the intervening time as the intangible cost(s) of stillbirth.

An exploration of the intangible costs of stillbirth will add to the evidence of the economic costs and consequences of stillbirth allowing a more complete appreciation of the burden of stillbirth. It is hypothesised that a realistic total cost will inform decision making on health resource allocation to prevent stillbirth or its adverse consequences.

Building on the earlier quantitative study by Mistry et al. [24] we carried out a synthesis of quantitative and qualitative studies to explore the evidence on the adverse consequences of stillbirths for parents and families. The main objectives for this study were to i) identify these consequences and ii) describe their economic implications in order to identify the key areas of impact that could be quantified using a questionnaire. These objectives were addressed from the time of diagnosis of fetal death up to care in the subsequent pregnancy.

## Methods

The literature search followed the Centre for Review and Dissemination (CRD) guidelines [25] and was reported in accordance with the PRISMA guidelines [26]. A background scoping search was done in June 2014 to identify key concepts, similar studies and research gaps in the economic analysis of stillbirth.

### Inclusion criteria

Papers were included if: the participants were mothers or/and fathers who had experienced stillbirth, the intervention/exposure was stillbirth and the outcome was the negative consequences of stillbirth. The study design included both qualitative and quantitative studies. The review was restricted to studies carried out in high-income countries (defined as the Organisation for Economic Co-operation and Development (OECD) member countries) and English language articles published in peer-reviewed journals. Papers published prior to 2000 were also excluded for pragmatic reasons as they may not reflect current experiences of parents.

### Search strategy

**Fig. 1:**
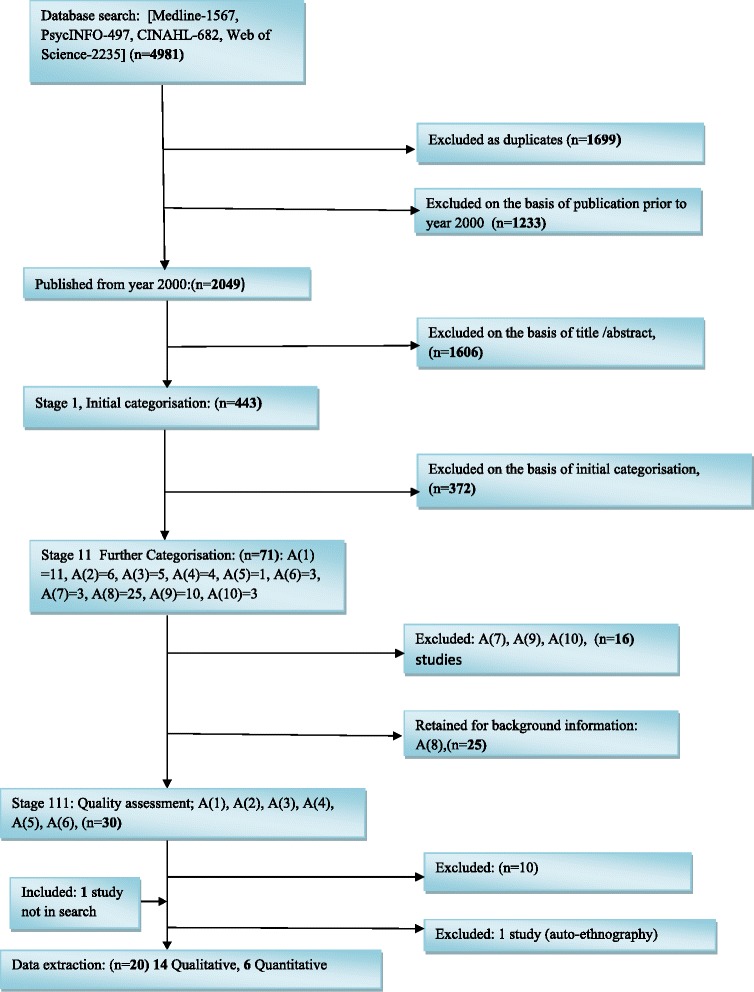
Flow diagram of papers through the studies

Four electronic databases: MEDLINE, PsycINFO, CINAHL and Web of Science (WoS) were comprehensively searched in June 2014 (Table 1). The reference lists of key papers were hand-searched to identify other relevant studies. The results were managed with Refworks reference manager database [27] and duplicates were removed both electronically and manually. Studies relevant to the review were selected in a three-stage process using established methods [28]. Initially, on the basis of title and abstracts, articles were screened and classified into seven groups (A to G) (Additional file 1). Second, full texts of potentially relevant studies were read and classified further (Additional file 2). The search strategy resulted in 4981 citations of which 1699 were duplicates. A flow diagram of the studies identified, selected, excluded or retained is shown in Fig. 1.

**Table 1:** MEDLINE search terms and results: using the Boolean logic terms “OR” and “AND”, these search term sets were used in strategy

#	Search terms	Results
**1**	Stillbirth$.mp. or Stillbirth/	8151
**2**	Fetal death$.mp. or Fetal Death/	25,887
**3**	Perinatal death$.mp.	3280
**4**	Perinatal loss$.mp.	486
**5**	Pregnancy loss$.mp.	4033
**6**	1 or 2 or 3 or 4 or 5	36,916
**7**	Psycho* effects.mp.	5108
**8**	Grief.mp. or exp Grief/	8957
**9**	Pain/px [Psychology]	12,474
**10**	Stress, Psychological/	88,636
**11**	Suffering.mp. or Stress, Psychological/	180,766
**12**	“Costs and Cost Analysis”/ or “Cost of Illness”/ or intangible costs.mp.	59,734
**13**	7 or 8 or 9 or 10 or 11 or 12	262,049
**14**	6 and 13	924
**15**	Cost$.mp. or “Costs and Cost Analysis”/	414,970
**16**	Economic$.mp.	203,888
**17**	15 or 16	563,572
**18**	6 and 17	1027
**19**	6 and 13 and 17	79
**20**	Limit 19 to (english language and humans)	**63**
**21**	Limit 18 to (english language and humans)	**761**
**22**	Limit 14 to (english language and humans)	**743**

Highlighted figures indicate final search terms results

A narrative review was conducted for the quantitative studies. Next, a quality appraisal was conducted for the qualitative papers and data were extracted from the papers for a synthesis. Finally, overarching themes from the qualitative synthesis were used to develop a proposed questionnaire for the quantification of intangible costs.

### Narrative synthesis

Data on aims, participants, outcome measured, measuring tools used and selected results for each quantitative study were extracted, tabulated and compared narratively.

### Quality assessment of selected studies

To evaluate the relevance of papers to the synthesis, a quality assessment of the qualitative studies was undertaken (Additional file 3) using a modified version of the Critical Appraisal Skills Programme (CASP) checklist [29] as used in previous qualitative syntheses [30, 31].

### Qualitative synthesis

The qualitative synthesis was carried out using meta-ethnography [32] as adapted to research by Britten et al. [33]. Meta-ethnography was used ‘to develop an inductive and interpretive form of knowledge synthesis’ [32]. Like meta-analysis, meta-ethnography uses many practical studies but, unlike meta-analysis, the sample is purposive and not exhaustive because the aim is to interpretatively integrate studies and not to predict them [33]. It has been successfully used in studies [30, 33] to achieve a deeper level of explanation than can be obtained using a narrative literature review [34].

The papers were carefully read by two reviewers (CO and LJ) in order to determine the central concepts, and the details of the study participants, aims and methods were tabulated to serve as a context for interpreting and explaining each study. Next, the main ideas and quotes for each study were identified and tabulated. To explicitly show how the concepts correlated with each other, a grid was created and the concepts of each paper placed in it. Using ideas developed by Schutz [33], first and second order constructs were developed. First order constructs are the original words of the participants while second order constructs are the researcher’s interpretation of those ideas [35].

Using reciprocal translation analysis (RTA) [32, 34], relationships between concepts emerging from the different studies were considered, and similar concepts and theories identified. Finally, in order to interpret meanings within the individual studies, overarching themes were developed.

### Development of proposed questions for a questionnaire

The themes from the qualitative synthesis were prioritised in terms of the frequency of their occurrence in the identified studies. Themes with similar ideas were merged into sections. In an attempt to move towards quantifying the evidence collated in the qualitative synthesis, questions are proposed, which could be developed for use in a large scale survey to provide this quantification. Finally, the questions developed from the sections were refined and related themes amalgamated to generate a proposed questionnaire.

## Results

### Search results

Six quantitative and 14 qualitative studies were selected for the review and synthesis.

### Narrative review of quantitative studies

Among the six quantitative papers, (Additional file 4) four focused on the effects of stillbirth on mothers only [36–39], while the others dealt with fathers/couples [40] and couples only [41]. Three of the studies were in Sweden [36, 37, 41] two were in the UK [38, 40] and one was multi-national [39]. Five studies used validated scales to quantify anxiety and depression among participants. In addition to the use of certified tools, three studies used questionnaires [36, 40] or interviews [38] to gather demographic data and information on experiences of stillbirth (Additional file 5).

The results (Table 2) show that the long-term anxiety scores and depression scores were higher in women that have experienced stillbirth than those with live births as was the case for fathers who have experienced stillbirth [36, 39]. Surkan et al. [37] found higher levels of depressive symptoms in mothers who were not allowed to hold their stillborn child for long enough compared to those who did. Turton et al. [40] found that these symptoms continued into the subsequent pregnancy and delivery. Depression level, state anxiety, trait anxiety and PTSD were all found to be higher in fathers that have experienced stillbirth [40]. The effect of stillbirth on siblings in a subsequent pregnancy was measured by Turton et al. [38]. Although no significant effect was found in children, they found that the maternal perception of its impact on their children was grossly inflated.

**Table 2:** Results of quantitative studies

Lead author (year)	Selected results	Author’s conclusion
Radestad (2001) [36]	Mean anxiety score: cases; 36.4, controls; 34.8	Slightly higher anxiety level in women with stillbirth compared to those without stillbirth
Turton (2006) [9]	Within-couple analysis (negative scores indicate higher levels in mothers), PTSD(−6.63), State anxiety (−4.42), Trait anxiety (−1.41)	Among couples with stillbirth, mothers had higher level of PTSD and anxiety than fathers
		Compared to parents without stillbirth, parents with stillbirth had significant levels of depression, anxiety and PTSD
Saflund (2006) [41]	WB: Women/ Men, Higher NWB (*p* = ≤ 0.0001) Lower PWB (*p* = ≤ 0.010) Lower GWB (*p* = ≤ 0.001)	At 3 months post stillbirth; mothers scored significantly higher on NWB and lower on PWB and GWB than fathers
		None of the fathers was on sick leave whereas all mothers were on full or part-time leave
Surkan (2008) [37]	Relative risk of depressive symptoms, Not held baby long enough (RR 6.9, 95 % CI 2.4–19.8), Not pregnant within 6 months (RR 2.8. 95 % CI 0.9–8.4)	Depression in mothers post stillbirth is influenced by the length of time they spent with their stillborn and if pregnant again within 6 months
Turton (2009) [38]	No significant association	No evidence to suggest that siblings born after a stillbirth are clinically at risk for psychological problems
Cacciatore (2013) [11]	Anxiety, 41.3 %, mental distress, 42.3 %, depressive symptoms, 61.7 %	Scores elevated among mothers that blamed themselves for a stillbirth

### Qualitative synthesis

Of the 14 papers that met the quality assessment criteria, two drew from the same data [42, 43]. The studies were all published between 2001 and 2013, with four based in Sweden [44–47], three in the United States [42, 43, 48], two in the UK [49, 50] and one each in Norway [51], Japan [52] and Australia [53] while two studies were online [54, 55]. Seven of the studies used in-depth interview, two used focus group discussion while the rest used open-ended questionnaires.

A profile of the 14 studies used for the qualitative synthesis was developed (Additional file 6) and the themes and concepts identified (Additional file 7).

**Fig. 2:**
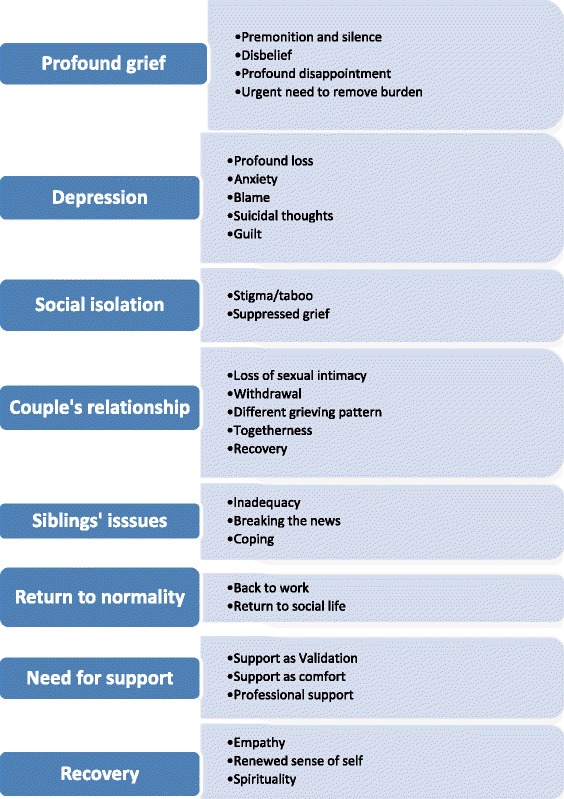
Themes and sub-themes identified in qualitative studies

Eight main themes were interpreted within these studies (Fig. 2): *profound grief*; *depression; social isolation; relationship issues; siblings’ issues; difficulty returning to normality; need for support* and *life changing event*. These will be discussed briefly.

#### Profound grief

This was a common theme in many of the studies especially on finding out that the baby had died. The initial shock was usually followed by great disappointment at the loss of the long-awaited arrival which created a huge gap in parents’ lives [44, 50, 55]. There was an urgent need to deliver the baby, amidst fear that the baby could harm the mother [44, 51]. The feelings of grief were found to linger for months and even years following stillbirth: *“The grief never fades away, I’m always aware of it. It comes on abruptly…”* ([44], pg 128).

#### Depression

Depression, a recurring theme in many studies [42, 44, 48, 50, 54, 55] was often debilitating in some cases leading to active or passive suicidal thoughts [42, 43]. Symptoms were long-lasting, often requiring medication and/or therapy: *“The months after were filled with therapy and medication for PTSD, anxious days and panicked nights”* ([42], pg 69). It was associated with a feeling of profound loss [42, 44, 48, 50, 52, 55] and anxiety even in the subsequent pregnancy [51, 55]. Depression due to blame [48, 49] and guilt [43, 55] was also common. Self-blame was reinforced by the socio-cultural beliefs of people around the participants: *“Once I started to say I was home-birth (and) I didn’t have any scans, I always got the feeling ……well that’s why your baby died, because you didn’t have a scan”* ([49], pg 479).

#### Social isolation

There is stigma/taboo surrounding stillbirth, making the grieving process difficult [48, 51]. The stigma arises from the misperception that it is the mothers’ fault; for example by smoking, drinking or misusing drugs. The stigma also affects fathers who are sometimes perceived as having genetic problems that could have led to the child’s death. Friends and colleagues are unwilling to discuss stillbirth and even society fails to recognize it as a valid grief: *“As a society, we really haven’t given it a place…”* ([48], pg 146). The societal pressure on males to be the stronger gender, isolates the fathers and their grief is often overlooked [44, 48, 53, 54]: *“You can’t say it was worse for…. because it was in her stomach; It is not. Obviously the baby was in there, but the bond and therefore, the loss is just as much”* ([53], pg 256). This pressure may also affect the relationship with the partner.

#### Relationship with partner

The negative effects of stillbirths on couples’ relationships were a recurring theme [44, 47, 52, 54]. Stillbirth led to a lack of sexual intimacy with most women losing interest in sex except for trying to conceive. The gender difference in the grieving pattern led to conflicts in the marriage and in some cases, its breakdown. There was reduced communication especially from fathers [47, 54], with feelings of loneliness and withdrawal from the relationship: "*Initially, my husband ‘shielded’ me from everything. After a very short time, he began refusing to acknowledge baby had existed and this put a great strain on us both. We eventually divorced”* ([54], pg 358).

#### Siblings’ issues

Studies [43, 45, 46, 52] described the difficulty amongst parents and health professionals in dealing with the siblings of the stillborn child. The grief of the children ranged from tears, guilt and sleep disorders in younger ones: *“The sibling talked loudly in her sleep, ‘of course I have a sister, although she is dead’”* ([46], pg 155), to silence and isolation in the older siblings: *“One of my children keeps his feelings to himself, but he says that he often thinks about what happened”*. ([45], pg 156). Most mothers were hounded by feelings of maternal inadequacy and in some cases the fathers took over the role of the main caregiver.

#### Difficulty returning to normality

Most studies reported that men usually, went back to work earlier than women [44, 47, 52, 54]. The perceived quick recovery by men led to resentment by some women on having to grieve alone: *“My husband went back to work quickly and seemed happy to do so…and I was left on my own”* ([47], pg 671). Although fathers were quicker to return to work, their grief did not go away: *“When I went back to work, it felt both good and awful; sometimes I just wanted to get away…..the grief never fades away, I’m always aware of it”* ([44], pg 128). A common theme was the difficulty in getting back to one’s previous social life which compounded parents’ social isolation: *“I didn’t go out socially for 6 months. My husband went to one get-together 3 months after our son’s death and came back within half an hour”* ([54], pg 361).

#### Need for support

A recurring theme in nearly all the studies [42–44, 48, 50, 51, 54, 55] is the importance of support, from the partner, friends, other bereaved persons, religious organizations or health professionals. Support was seen as a validation of grief especially when it came from people with similar experience [42, 54, 55]: *“having people acknowledge her life and death and her impact on my family, helped me to know that I’m not crazy”* ([42], pg 67). Professional support although available was felt to be limited and lacking in experience: “*There is a strong need to find a therapist that has experienced the loss….the death of a child….”* ([42], pg 71). Many couples reported that the support should be in place for the longer term.

#### Life changing event

Many studies described stillbirth as a life changing event for many parents [42, 48, 54]. Themes like empathy (reaching out to other grieving parties), renewed sense of self and a change in spirituality were a means of recovery. There was a change in the way they viewed life and themselves: *“My child’s death has changed me to be a more sensitive person to other’s feelings…..”* ([42], pg 68).

### Proposed questions to capture intangible costs and consequences

**Fig. 3:**
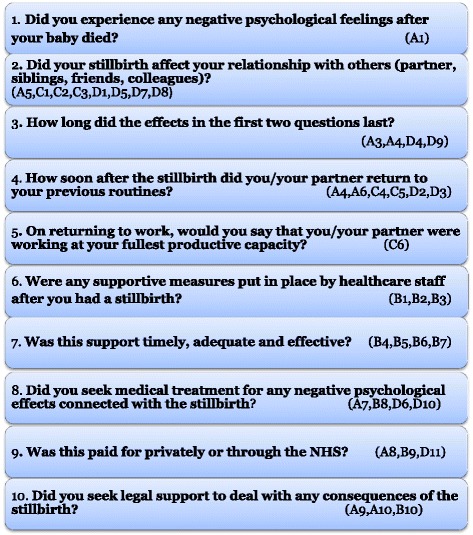
Top 10 questions and represented sections

The merging of themes with similar ideas resulted in four sections and after refining and blending similar questions, 10 questions were proposed to cover the breadth of intangible costs and they focus on depression/profound grief, need for support, social isolation/return to normality and couples’ relationship/siblings' issues (Fig. 3 and Additional file 8).

Among the eight themes from the meta-ethnography, the outstanding theme that emerged from nearly all the studies was ‘*depression’*. This also came out strongly in the narrative review. This theme was followed closely by ‘*need for support’.* The next two most common themes were ‘*profound grief’* and ‘*social isolation’*. Three themes: ‘*couples’ relationship’, ‘siblings’ issues’* and ‘*return to normality’* seemed to be of relatively equal importance. However, two of these themes *(couples’ relationship* and *siblings’ issues)* have the same connotation and seemed to point towards similar issues relating to impacts on the family. Less common was the theme of ‘*recovery’* which is arguably an intangible benefit and therefore, is less relevant to the aim of the study.

## Discussion

We aimed to find studies that identified the consequences of stillbirth, and their duration and impact so that they could be quantified and moved from intangible to tangible costs. A number of studies reported these negative feelings but most did not explicitly report the duration or their impact on quality of life. However, several studies [42–45, 47, 49, 54] mentioned that the feelings continued for months and even for years indicating prolonged effects.

The overarching themes of profound shock and depression were consistent with results from the narrative review which showed that the high level of depression, anxiety and PTSD after a stillbirth, persisted for months [41] and up to the subsequent pregnancy [40]. Depression if unrecognized or untreated can lead to self-harm and in some cases, suicide [56]. The underlying implication is that the reduced quality of life due to these symptoms will require long-term therapy and treatment, either funded privately or by state-supported health services. Hence, these intangible costs to parents potentially involve financial costs for them and for society at large. Such mental health problems can be compounded by feelings of isolation due to the associated stigma of stillbirth limiting the emotional and social functioning of affected parties both within and outside the family unit. Feelings of isolation could also manifest as depression and physical symptoms [56], resulting in therapy being sought at a cost.

The synthesis also showed that stillbirth could cause relationship issues among couples leading to separation and divorce. Gender differences in grieving may lead to misunderstandings which inadvertently affect family functioning and relationships [57, 58]. Relationship failure can then lead to hardship, ill health, low income and poor satisfaction with life (Gulson, 1976 in [59]). Statistics suggest that the odds of divorce are greater among women who have experienced stillbirth than those who had a live birth [60, 61]. Divorce incurs costs, both to the parties involved and to society; the resulting effects on families ripples over to the children who may experience depression or difficult behaviour. Even in the absence of parental relationship breakdown, siblings may be neglected leading to behavioural problems [59]. In such cases, children may require healthcare interventions such as medication and counseling, again translating the intangible costs of stillbirth into direct healthcare costs. Yet in contrast, some couples were able to make sense of their own life and their bereavement strengthened their relationship which needs to be taken into consideration [62].

The synthesis showed that men returned to work earlier than women with all fathers returning to work and most mothers either on full or part-time leave by the third month after a stillbirth [41]. The concept of early return to work amongst the fathers could be viewed as a positive aspect as the indirect cost from loss of productivity is reduced, but despite an early return to work, fathers continue to grieve and take frequent breaks during work to grieve alone. Thus fathers are frequently unable to function to their full productive capacity at work, a concept known as ‘presenteeism’ and therefore incur costs to society.

The importance of formal and informal support cut across all aspects of the studies, with many parents feeling they needed expert support and reporting that support was limited. The Royal College of Obstetricians and Gynaecologists (RCOG) guidelines for care of families following a stillbirth, acknowledges the possibility of the need for psychological care but there is no practical advice on how healthcare professionals can support parents [63]. The isolation experienced by parents emphasizes the need for a more formal support in the form of counseling, therapy and support groups. The observation that poor levels of social support are associated with prolonged grief [64] highlights the importance of understanding the intangible costs of stillbirth as provision of support incurs costs but could potentially save money by preventing mental-health problems.

Some sub-themes such as empathy, a better relationship with one’s partner and a renewed sense of self reflect intangible benefits rather than costs. Moreover, this stage usually, did not happen overnight and was preceded by months and years of grief: *“…I spent so many years trying to find that ‘something’ that will give me peace”* ([42], pg 72). So these “benefits” may still result in a net cost to the individual and society.

The main strength of this paper is that it is the first to identify the probable main sources of the intangible costs of stillbirth. The only indirect evidence that presently exists is in studies that have described the psychosocial consequences of stillbirth. A second strength is the comprehensive and systematic attempt to identify relevant studies. The inclusion of both quantitative and qualitative studies conducted in the last 15 years allowed a deep assessment of the issues highlighted by these studies. The search strategy and the three-stage selection/exclusion process were wide and detailed. The use of meta-ethnography to interpret first-order constructs and produce new understandings resulted in a deeper insight and development of concepts than that obtained in a narrative literature review alone. Finally, the study addressed the research gap identified in a previous study on costs of stillbirth [24].

As expected in any study, there were also limitations. Firstly, the study did not assess the relevance of the findings of the synthesis. A possible way of checking this would be to present it in a questionnaire form to population groups that have experienced stillbirth. However, by proposing questions that could be asked in prospective large scale surveys, the study developed a framework to guide future studies. A second limitation was the presentation of these questions in the words of the authors alone. Thus for a questionnaire, the questions will have to be refined by experts from this patient group to ensure the utmost sensitivity in the language used. Finally, there are controversies surrounding the inclusion of intangible costs in costing estimates [15, 19, 65, 66], thus the majority of economic evaluations include only their qualitative discussion [23]. However although intangible costs are difficult to quantify, previous studies from other fields have attempted this by various methods [15, 65–71]. However, such quantification was not attempted here.

This study has important implications for health economists; maternity care providers and policy makers. Firstly, the findings justify the rationale for including intangible costs in economic evaluations of stillbirth by revealing their potentially significant contribution to the total costs. Thus, the common approach of estimating only direct and indirect costs is likely to miss relevant aspects of the total disease burden. It has been said that intangible costs are difficult to quantify so are not included in most studies. However, some studies have used recognised approaches such as “willingness-to-pay” (WTP) methods to place a monetary value on these costs. WTP is a complex method requiring specialized expertise in designing and implementing surveys and its use in this area is limited [19, 23].

Secondly, the intangible costs, in addition to direct/indirect costs of stillbirth could be compared more realistically to the costs of other maternal or fetal outcomes such as live-births and premature births. This comparison will be useful for policy makers to decide on resource allocation to these sectors. Thirdly, after a stillbirth, supportive measures could be instituted at once; from the diagnosis, into and beyond the subsequent pregnancy to offset some of the intangible costs. Ultimately, evidence-based guidelines on how to practically support families after a stillbirth need to be developed. To address this need the policy emphasis needs to be directed to providing more funds towards development of interventions to reduce the adverse psychological effects of stillbirth on families. In addition, a true appreciation of the costs of stillbirth will allow an appropriate cost-benefit assessment of interventions to prevent stillbirth which are presently impossible.

There is undoubtedly a need for more economic evaluation studies to be conducted on stillbirth. The area is still sparsely researched which has not allowed us to consider the impacts on health-service delivery or on health-care professionals. One suggestion for future studies would be to put a monetary value on these intangible costs. Furthermore, similar studies could also be carried out in low and middle income countries.

## Conclusions

The study has shown that stillbirths have a wide reaching and long-lasting impact with far-reaching economic implications. Considering the significant numbers of stillbirth worldwide, the large number of families affected and the long-lasting nature of its impact, it is surprising that stillbirths still attract relatively little attention from policy makers. Therefore, exploring the intangible costs associated with stillbirth is important to emphasize its burden and inform policy and decision making.

## Additional files

Additional file 1:
**Stage 1 grouping.** (DOC 22 kb) Additional file 2:
**Stage 2 grouping.** (DOC 22 kb) Additional file 3:
**Results of the quality assessment using the Critical Appraisal Skills Programme (CASP) tool.** (DOC 53 kb) Additional file 4:
**Characteristics of quantitative studies.** (DOC 33 kb) Additional file 5:
**Measures and tools for quantitative studies.** (DOC 41 kb) Additional file 6:
**Profile of the qualitative studies used in the synthesis.** (DOC 49 kb) Additional file 7:
**Qualitative synthesis: themes, subthemes, constructs and included papers.** (DOC 72 kb) Additional file 8:
**Merging of themes.** (DOC 29 kb)

## Abbreviations

BDI: Beck depression inventory
CASP: Critical appraisal skills programme
CES-D: Centre for Epidemiological Studies Depression Scale
CINAHL: Cumulative index to nursing and allied health literature
CRD: Centre for Reviews and Dissemination
DSM-IV: Diagnostic and statistical manual of mental disorders 4th edition
EPDS: Edinburgh postnatal depression scale
FGD: Focus group discussion
GWB: General well being
GRIMS: Golombok-Rust Inventory of Marital State
HSCL: Hopkins symptom checklist
MEDLINE: Medical literature analysis and retrieval system online
MESH: Medical subject headings
NWB: Negative well being
PRISMA: Preferred reporting items for systematic reviews and meta-analyses
PTSD: Post-Traumatic stress disorder
PWB: Positive well being
RTA: Reciprocal translation analysis
RCOG: Royal College of Obstetricians and Gynaecologists
SCID: Structured clinical interview for DSM-IV
SDQ: Strengths and difficulties questionnaire
STAI-T: State-trait anxiety inventory
WASI: Wechsler abbreviated scale of intelligence
WBQ-12D: Well being questionnaire-12 dimension
