# Supplementary material for: Exploring the intangible economic costs of stillbirth
Source: BMC Pregnancy Childbirth. 2015 Sep 1;15:188. doi: 10.1186/s12884-015-0617-x (PMC4556317; doi:10.1186/s12884-015-0617-x)
Supplement: Additional file 1: — Stage 1 grouping. (DOC 22 kb) [file 12884_2015_617_MOESM1_ESM.doc]

Additional file 1: Stage 1 grouping

1. The study reports information that can be potentially defined as intangible costs (pain/suffering) of stillbirth;
2. The study reports information that can be potentially defined as intangible costs (pain/suffering) of perinatal (stillbirth or early neonatal) death;
3. The study reports the indirect costs (productivity loss) associated with stillbirth;
4. The study reports the direct costs associated with stillbirth;
5. The study may have background information but does not fall into (A), (B), (C) or (D);
6. The study reports information that can be potentially defined as intangible costs of stillbirth in countries outside the OECD;
7. The study has no relevance to quantifying the intangible costs of stillbirth.
