# Supplementary material for: Exploring the intangible economic costs of stillbirth
Source: BMC Pregnancy Childbirth. 2015 Sep 1;15:188. doi: 10.1186/s12884-015-0617-x (PMC4556317; doi:10.1186/s12884-015-0617-x)
Supplement: Additional file 2: — Stage 2 grouping. (DOC 22 kb) [file 12884_2015_617_MOESM2_ESM.doc]

Additional file 2: Stage 2 grouping

1. Psychological effects in mothers after stillbirth;
2. Psychological effects in fathers after stillbirth;
3. Psychological effects in parents after stillbirth;
4. Psychological effects in siblings and grandparents after stillbirth;
5. Psychological effects in families after stillbirth;
6. Psychological effects in a subsequent pregnancy after stillbirth;
7. Psychological effects of stillbirth among healthcare professional;
8. Support for families with stillbirth;
9. Coping measures for mothers after stillbirth;
10. Does not fall into any of the classes.
