# Supplementary material for: Exploring the intangible economic costs of stillbirth
Source: BMC Pregnancy Childbirth. 2015 Sep 1;15:188. doi: 10.1186/s12884-015-0617-x (PMC4556317; doi:10.1186/s12884-015-0617-x)
Supplement: Additional file 3: — Results of the quality assessment using the Critical Appraisal Skills Programme (CASP) tool. (DOC 53 kb) [file 12884_2015_617_MOESM3_ESM.doc]

## Additional file 3: Results of the quality assessment using the Critical Appraisal Skills Programme (CASP) tool

| **#** | **Study** | **Clear Statement of the aims of the research?** | **Qualitative methodology appropriate?** | **Research design approp. ?** | **Recruitment started approp. ?** | **Data collected addressed the research issue?** | **Relationship between researcher and participant adequtely considred?** | **Ethical issues addressed?** | **Data analysis sufficiently rigorus** | **Findings clearly stated?** | **Value of research** | **Notes** |
| --- | --- | --- | --- | --- | --- | --- | --- | --- | --- | --- | --- | --- |
| 1 | Samuelsson (2001) | Y | Y | ? | Y-But slightly flawed | Y | N-No information | Y-Ethical approval | Y | Y | SAT |  |
| 2 | Trulsson (2004) | Y | Y | Y | Y | Y | N | Y | DK- No information | Y | ? |  |
| 3 | Cacciatore (2007) | Y | Y | Y | Y | Y | Y | Y-Anonymity considered | Y- But not much information | Y | ? | Though dealt with support, had useful information on grief and suffering |
| 4 | Cacciatore (2008) | Y | Y | Y | Y | Y | N-No information provided | N- No information given | Y | Y | KP |  |
| 5 | Cacciatore (2010) | Y | Y | Y | Y | Y | Y | Y- Ethical approval | Y- But not much information | Y | KP |  |
| 6 | Erlandsson (2010) | Y | Y | Y | Y | Y | Y | Y-Anonymity guaranteed | Y- both quantiative and qualitative | Y | SAT | Questionnaire developed using FGD to identify study-specific questions |
| 7 | Yamazaki (2010) | Y | Y | Y | Y | Y | Y | Y | Y | Y | KP |  |
| 8 | Avelin (2011) | Y | Y | Y | Y | Y | N-No information | Y-Ethical approval | Y | Y | SAT |  |
| 9 | Bonnette (2011) | Y | Y | Y | Y- Difficulty acknowledged | Y | N-No information | Y-Ethical approval | Y | Y | SAT |  |
| 10 | Kelly (2012) | Y | Y | Y | N | Y | N-No information | Y-Ethical approval | Y | Y | SAT |  |
| 11 | Murphy (2012) | Y | Y | Y | N –But very detailed information on difficulties | Y | Y | Y- Ethical approval | Y- detailed information | Y | KP |  |
| 12 | Weaver-Hightower (2012) | Y | Y | N-None used | N- No recruitment | N.N/A | N-N/A | N | N |  | ? | Auto-ethnography based on personal experience. Has useful information by incorporating the experiences of others |
| 13 | Avelin (2013) | Y | Y | Y | Y | Y | Y | Y-Ethical approval obtained | Y-Qualitative and quantitative | Y | SAT |  |
| 14 | Downe (2013) | Y | Y | Y | Y | Y | Y | N-No information | Y | Y | KP |  |
| 15 | Lee (2013) | Y | Y | Y | Y | Y | Y | N-No information | Y | Y | SAT |  |
