# Supplementary material for: Exploring the intangible economic costs of stillbirth
Source: BMC Pregnancy Childbirth. 2015 Sep 1;15:188. doi: 10.1186/s12884-015-0617-x (PMC4556317; doi:10.1186/s12884-015-0617-x)
Supplement: Additional file 4: — Characteristics of quantitative studies. (DOC 33 kb) [file 12884_2015_617_MOESM4_ESM.doc]

## Additional file 4: Characteristics of quantitative studies

| **#** | **Lead author (date)** | **Aim of study** | **Participants** | **Further information on methods** |
| --- | --- | --- | --- | --- |
| **1** | Rảdestad (2001), Sweden | To investigate if obstetric care during stillbirth had psychological effects in the long-term | 759 women. 380 who had given birth to stillbirth (cases) and 379 had not (controls) | Questionnaire designed by researcher in a pilot study |
| **2** | Turton (2006), United Kingdom | To assess the psychological states of fathers and compare within-couple effects in the subsequent pregnancy | 38 pregnant couples with previous stillbirth and 38 pair-matched controls | Cohort |
| **3** | Saflund and Wredling (2006), Sweden | To investigate differences between couples and their stillbirth experiences | Recruited over 12 months. 22 couples with stillbirth experience | Questionnaire developed by researcher based on clinical experience and FDGs with fathers with stillbirth experience |
| **4** | Surkan (2008), Sweden | Effect of bonding with baby on maternal mental health |  |  |
| **5** | Turton (2009), United Kingdom | Effect of stillbirth experience on maternal perception of children born in the subsequent pregnancy | Mothers with stillbirth experience with next-born children 6-8 yrs  52 mothers versus 51 mother-child dyads | Case-control study |
| **6** | Cacciatore (2013), International (US, UK, Australia and Canada) | The effect of self-blame on maternal mental health after a stillbirth | Women who responded to an online questionnaire.  2,332 women | Questionnaire developed by researchers |
|  |  |  |  |  |
