# Supplementary material for: Exploring the intangible economic costs of stillbirth
Source: BMC Pregnancy Childbirth. 2015 Sep 1;15:188. doi: 10.1186/s12884-015-0617-x (PMC4556317; doi:10.1186/s12884-015-0617-x)
Supplement: Additional file 5: — Measures and tools for quantitative studies. (DOC 41 kb) [file 12884_2015_617_MOESM5_ESM.doc]

## Additional file 5: Measures and tools for quantitative studies

| **Study** | **Measure** | **Tools** |
| --- | --- | --- |
| **Radestad (2001)** | Anxiety | State-Trait Inventory (STAI-T) |
|  | Depression | Centre for Epidemiological Studies Depression Scale (CES-D) |
|  |  | Questionnaire |
| **Turton (2006)** | Depression | Beck Depression inventory (BDI), Edinburgh Postnatal Depression Scale (EPDS) |
|  | Anxiety | STAI-T |
|  | Tendency anxiety | STAI-T |
|  | PTSD | PSTD-I Interview |
|  | Marital Satisfaction | GRIMS |
|  |  | Questionnaire |
| **Saflund (2006)** | Psychological wellbeing | Well being Questionnaire (WBQ-12D) |
|  |  |  |
|  |  |  |
|  |  |  |
| **Surkan (2008)** | Depression | CES-D |
|  |  |  |
|  |  |  |
|  |  |  |
| **Turton (2009)** | Maternal psychological morbidity | Structured clinical interview for DSM-IV |
|  | Child emotions | Strengths and Difficulties questionnaire (SDQ) |
|  | Child verbal performance and IQ | Wechsler Abbreviated Scale of Intelligence (WASI) |
| **Cacciatore (2013)** | Anxiety | Hopkins Symptoms Check List (HSCL) (25 items) |
|  | Depression |  |
