# Supplementary material for: Exploring the intangible economic costs of stillbirth
Source: BMC Pregnancy Childbirth. 2015 Sep 1;15:188. doi: 10.1186/s12884-015-0617-x (PMC4556317; doi:10.1186/s12884-015-0617-x)
Supplement: Additional file 6: — Profile of the qualitative studies used in the synthesis. (DOC 49 kb) [file 12884_2015_617_MOESM6_ESM.doc]

## Additional file 6: Profile of the qualitative studies used in the synthesis

| **#** | **Study** | **Study aims** | **Further information on studies** | **Participant detail** | **Time after stillbirth** | **Methods** | **Further information on Methods** | **Analysis** |
| --- | --- | --- | --- | --- | --- | --- | --- | --- |
| 1 | Samuelsson et al (2001), Sweden | To describe fathers’ experience of losing a child | Fathers as the forgotten mourners | 11 men aged 31-46 | 5 to 27 months after stillbirth | In-depth interviews | Interview of 25 minutes to 2 hours, at home or hospital | Phenomenology |
| 2 | Trulsson and Radestad (2004), Norway | To investigate women’s experience before and during baby’s death diagnosis, the delivery, and shortly after delivery | How having a stillborn affects parents both as individuals and as a couple | 12 Women who gave birth between July 1999 and July 2000 | 6 to 18 months after stillbirth. | Interview | Interviews of 90 minutes average | Phenomenology |
| 3 | Cacciatore and Bushfield (2007), Worldwide | To explore the experiences that helped bereaved mothers to move through their grief | The lack of care for stillbirth despite progress in social work | 47 women, aged 19-51. 38 European Americans, 3 African Americans, 3 Latinos, 2 others and 1 Asian  Recruited by purposive and snowball sampling through non-profit organisations that provide bereavement care | Past one year (n=10); One to two years (n=10) 2=5 years (n=17) 5 to 10 years (n=7) more than 10 years (n=3) | Open-ended Questionnaire | Self-administered questionnaire on current feelings and emotions | Phenomenology (a method for gaining insight into the lived experiences) |
| 4 | Cacciatore (2008), Worldwide | To examine bereaved parents perception and coping measures of their child’s death. | Effect of stillborn on the parents both as individuals and as a couple | Couples recruited through parental grief conference and online=based parental support organisations.  74 participants (55 at conference, 19 online) | None specific (very recent to several years) | Open ended questionnaire | Questionnaire informed by empirical research on infant death | Grounded theory |
| 5 | Cacciatore (2010), United States | To examine how a woman’s stillbirth experience affects her both as an individual and as a member of the family system. | Are there any long-lasting effects of SB?  If so, what variables determine these changes | Women who have experienced stillbirths  47 women  (same data as no.3) | Less than 1 year to more than 10 years (n=3) | Open ended questionnaires | Questionnaire was part of mixed method study on support after SB | Phenomenology |
| 6 | Erlandsson (2010), Sweden | To examine parents’ views of ways in which siblings say goodbye to and mourn a stillbirth brother or sister and how parents support them | Provide insight on increasing hospital staff sensitivity to experience of the whole family | 16 parents of siblings to a stillborn child one year after the stillbirth (12 mothers and 4 fathers) recruited over 1 year | 1 year | Questionnaire | Developed by researcher based on a literature review and clinical experience of meeting parents of stillborn babies | Content analysis |
| 7 | Yamazaki (2010), Japan | To define the implication of stillbirth in the lives of women | Description of how the stillborn continues to exist in her daily lives | 17 women aged 28 to 38 years Sample selected by nonprobability convenience sampling | Between 1 and 6 year after stillbirth | In-depth semistructured interviews | Interviews of two to three hours | Grounded theory |
| 8 | Avelin (2011), Sweden | To investigate parents’ perspectives of their parenting capacity and siblings needs after a stillbirth. | The interplay between focusing on their grief and that of their other children | Parents with other children who have experienced a stillbirth  27 parents | Mean of 6 years after stillbirth (Range 1-22 years) | 6 FGDs (3-7 participants per group) | FGDs conducted between 2008 and 2010 lasting 90 to 120 minutes | Qualitative Content Analysis |
| 9 | Bonnette and Broom (2011), Australia | To explore the interplay of gender identity and concepts of fathering. | The experiences of loss, grief and male identity. | 12 men aged 28-54 who have experienced stillbirth. Recruited over 6 months using a combination of snowball and purposive sampling. | Within 3 years | Exploratory in-depth Interviews | The interviews explored a range of issues including: their lived experiences of stillbirth; how they  perceive themselves in the context of their unborn and then stillborn child  (i.e. as fathers); and, the experience and expression of grief | Interpretative |
| 10 | Kelly and Trinidad (2012), United States | To investigate parents’ experiences of stillbirth and the bereavement process with focus on psychological and emotional impact.  To understand doctors’ views and beliefs about stillbirth | Stigma, and profound grief of stillbirth | Parents who have experienced stillbirth.  3 parents groups (however 2 of the groups consisted entirely of mothers) |  | 3 semi-structured Focus Group Discussions with fathers and 2 FGDs with obstetricians-gynaecologists | Data collected in the course of a needs-assessment study (GAPPS) | Thematic discourse analysis |
| 11 | Murphy (2012), UK | To describe how ideas and views about women behaviour during pregnancy affect those who suffer stillbirth. | Stigma, Blame and the moral mother. | Couples and mothers who have experienced stillbirth. 10 couples and 12 mothers | 3 months to 20 years | In-depth interviews |  | Grounded theory (Strauss and Corbin) |
| 12 | Avelin (2013), Sweden | To describe parents’ grief at 3 months, 1 year and 2 years after a stillbirth and its effects on their relationships. | The experience is framed by the gender roles around parenthood | 33 mothers & 22 fathers (3 mths after SB)  31 mothers & 18 fathers (1 yr after SB)  26 mothers & 15 fathers (2 yrs after SB) | 3 months, 1 year and 2 years | Questionnaires | Questionnaires (developed by the researcher based on literature review and FGDs with fathers that have experienced stillbirth) | Content analysis |
| 13 | Downe et al (2013), UK | To identify parents view of interactions with health staff after a stillbirth experience | The experience of stillbirth in the UK | 22 families. Sample recruited from respondents to a previous survey | Between 1 to 9 years | In-depth interview | Face-to-face or telephone interview | Grounded theory (Constant comparative technique) |
| 14 | Lee (2013) | To understand womens’ thoughts about making decisions and the experience of the subsequent pregnancy following stillbirth | Development of a model | Sample selected from participants in a previous study  11 women aged 26 to 4 years | 6 to 10 months after stillbirth | In-depth interview |  | Modified grounded theory |
