# Supplementary material for: Exploring the intangible economic costs of stillbirth
Source: BMC Pregnancy Childbirth. 2015 Sep 1;15:188. doi: 10.1186/s12884-015-0617-x (PMC4556317; doi:10.1186/s12884-015-0617-x)
Supplement: Additional file 7: — Qualitative synthesis: themes, subthemes, constructs and included papers. (DOC 72 kb) [file 12884_2015_617_MOESM7_ESM.doc]

Additional file 7 Qualitative Synthesis: themes, subthemes, constructs and included papers

| **Over-arching theme** | **Sub –theme** | **Description of sub themes (2^nd^ order constructs)** | **First order constructs** | **Papers that included**  **Constructs** |
| --- | --- | --- | --- | --- |
| Profound Grief | Premonition and silence | Whether respondents felt the baby might die | It was like a curtain had been pulled down; it was trying. I didn’t believe it was true, it simply couldn’t be true. I saw on the ultrasound screen that the baby was not moving. In fact, I don’t know how I felt . . . shock, it’s impossible to describe it. I don’t know what could happen that could be worse. (Samuelsson et al, 2001, pg 125) | Samuelsson et al, 2001; Trulsson and Radestad, 2004; Downe et al, 2013; Lee et al, 2013. |
|  | Disbelief | It only happens to others | “There was a ‘stillbirth’ chapter in one of my pregnancy books. I skipped it.”  “You think this is something that only happens in third world countries, but not to us with our health care resources.” (Kelly and Trinidad, 2012, pg 3)  “I was just bowled over by the horror” (Downe et al, 2013, pg 5) | Samuelsson et al, 2001; Trulsson and Radestad, 2004; Yamazaki et al, 2010; Kelly and Trinidad, 2012; Downe et al, 2013; Lee et al, 2013. |
|  | Profound disappointment | The failed expectations of the baby’s arrival | “Emotionally everything had been geared towards having a baby and then there was a big hole, a baby-shaped hole which was much bigger than a baby.” (Lee et al, 2011, pg 434) | Samuelsson et al, 2001; Downe et al, 2013; Lee et al, 2013. |
|  | Urgent need to remove the burden | There was the fear that the dead baby in her body could harm her | I thought it was totally…I thought it was totally awful; I got this total feeling of dread; I didn’t want it. I just kept thinking: I’m alive, I lying here, aren’t I, and I’m alive, I can’t have a dead baby inside me, that just can’t be. My belly was a lump. I didn’t feel anything anymore (Trulsson and Radestad, 2004, pg 191) | Samuelsson et al, 2001; Trulsson and Radestad, 2004. |
| Depression | Profound loss | Grief following stillbirth is ambiguous | “(His) death has by far been the most devastating, life-changing event in my life.” (Cacciatore and Bushfield, 2007, pg 69)  “My saddest memories are also the ones I cherish” (Kelly and Trinidad, 2012, pg 143)  “The months after were filled with therapy and medication for post-traumatic stress disorder, anxious days and panicked nights. I am no longer whole. A part of me is gone forever-literally. Like a limb torn from a body. . .” (Cacciatore and Bushfield, 2007, pg 69) | Samuelsson et al, 2001; Cacciatore and Bushfield, 2007; Yamazaki, 2010; Kelly and Trinidad, 2012; Downe et al, 2013; Lee et al, 2013. |
|  | Anxiety | Pregnancy specific anxiety; the fear of another stillbirth | “I think the whole point is you lose the luxury to feel that bad things don’t happen to you, they happen to someone else.” (Lee et al, 2011, pg 434) | Trulsson and Radestad, 2004; Lee et al, 2013. |
|  | Blame | Blaming of self, God, and others | My Mum kept saying ‘You killed him, you killed him’. She kept saying ‘You didn’t know, you read all these books and you didn’t know what was happening’ …... And you know, for a long time I blamed myself.  Once I started to say that I was homebirth [and] I didn’t have any scans……I always got that feeling that there was this kind of, ‘Well that’s why your baby died, because you didn’t have a scan (Murphy, 2012, pg 479)  “Yes, depression, but more anger— anger at the medical system, and friends, family, and ourselves.” (Kelly and Trinidad, 2012, pg 144) | Kelly and Trinidad, 2012; Murphy, 2012. |
|  | Suicidal thoughts | Passive and active thoughts of self-harm | “A week or so after (he) died . . . I wanted to die. I never tried. I just wished I wasn’t here.” (Cacciatore, 2010, pg 140) | Cacciatore and Bushfield, 2008; Cacciatore 2010. |
|  | Guilt | Feelings of regret and guilt | “I have moments when I apologize for killing our daughter even though there was nothing I could do to stop it . . .” (Cacciatore, 2010,pg 140) | Cacciatore 2010; Downe et al, 2013; Lee et al, 2013. |
| Social isolation | Stigma/Taboo/Silence | Unwillingness of friends and colleagues to discuss the stillbirth | “I know it makes some people  uncomfortable, but I want to talk about my daughter” (Kelly and Trinidad, 2012, pg 145)  As a society, we really haven’t given it a place, so if somebody did have that experience, it was very quiet...(There isn’t an appropriate way to grieve a child that is stillborn, especially when people have never seen it (Kelly and Trinidad, 2012, pg 146) | Cacciatore 2010; Kelly and Trinidad, 2012. |
|  | Suppressed grief | Male gender expectations of being strong for the partner | You can’t say it was worse for Melissa because it was in her stomach; it is not. Obviously the baby was in there but the bond and therefore the loss is just as much. (Bonnette and Broom, 2011, pg 256)  . I think it was because she [wife] wasn’t there and I could lose it for a minute and not feel that I’m going to cause her to lose it even more. I felt like if I was with her and I lost it we would never recover  he was the first thing to make me aware of, you know, it’s alright for everyone patting Amber on the back and giving her a hug to see how she’s coping but how are you going Patrick? How are you personally going? He was the first person to bring it to the attention of maybe I’m struggling a little bit. | Samuelsson et al, 2001; Cacciatore, 2008; Bonnette and Broom, 2011; Kelly and Trinidad, 2013. |
| Couple’s Relationship issues | Loss of sexual intimacy | Loss of interest in sex except for trying to conceive | [There was a] major decrease [of sexual drive] on my end. Didn’t want to have sex unless we were going to [try to conceive] again . . (Cacciatore, 2008. Pg 359) | Cacciatore, 2008; Avelin, 2013. |
|  | Withdrawal | Feelings of loneliness and withdrawal in the relationships | “Initially, [my] husband ‘‘shielded’’ me from everything and everyone. After a very short time he began refusing to acknowledge baby had existed and this put a great strain on us both. We  eventually divorced” (Cacciatore, 2008. Pg 358)  ‘Sometimes I cannot understand how my partner does not need to think or talk about our stillborn child, especially when I think all the time of what happened (Avelin,2013, pg 671) | Cacciatore, 2008; Avelin, 2013. |
|  | Differences in grieving pattern | Conflict over differing styles of grieving and communication | “We recognize and respect that we grieve VERY differently. We have not grieved together much at all.”  (Cacciatore, 2008. Pg 359)  “I think I am still dealing with how to incorporate the loss into my life on a personal level. Whereas my husband seems less affected. There is a degree of disconnect between us. Emotionally  we are working to find each other again” (Cacciatore, 2008, pg 358) | Cacciatore, 2008; Yamazaki, 2010; Avelin, 2013. |
|  | Togetherness | Emotional bonding over grief | ‘To lose our baby brought us closer together in grief; we could talk and hold each other, our relationship as a couple has changed, we are closer and we have this unspoken bond’. (Avelin, 2013, pg 670) | Cacciatore, 2008; Avelin,2013. |
|  | Recovery | Resentment over ease of partner’s return to work | ‘My husband went back to work quickly, and seemed happy to do so…and I was left on my own ( Avelin 2013, pg 671) | Samuelsson et al, 2001; Avelin, 2013. |
| Siblings issues | Inadequacy | Feelings of incapability to take care of their other children especially by mothers | It was as if I had to split myself in two. One half had to be happy for Agnes’ sake. I actually had to be happy for her sake, be there for her, and then the other half that actually allowed me to be sad. (Mother of 2- and 15-year-old siblings, year of  stillbirth: 2007) (Avelin, 2011.pg 155) | Cacciatore, 2010, Erlandsson, 2010; Avelin, 2011. |
|  | Breaking the news | Did siblings meet their dead brother or sister | We became a little upset because we asked for help: What do we say to our son? But there was no-one that helped us. We had to deal with it ourselves. When Eric (the sibling) came, the first  thing he said when he saw the baby was ‘Why are her eyes closed?’ and I became very sad and started to cry, and he wondered why I was crying and then I turned for help to the staff present.  ‘What am I supposed to say now?’ But no one replied, so I told Eric that Saga (the stillborn) had been sick and that she was not alive. (Mother of 4-year-old sibling, year of stillbirth: 2008) (Avelin, 2011, pg 153) | Cacciatore, 2010; Erlandsson, 2010; Yamazaki, 2010; Avelin, 2011. |
|  | Coping |  | The sibling talked loudly in her sleep ‘of course I have a sister, although she is dead,’ and I felt that she was responding to the preschool teachers and their lack of acknowledgement of this fact. (Mother of sibling aged 2 years 6 months, year of stillbirth: 2007) (Avelin, 2011, pg 155) | Cacciatore, 2010; Erlandsson, 2010; Avelin, 2011. |
| Difficulty in returning to normalcy | Return to work | Gender difference in return to work | My] husband went back to work quickly—after about a week and a half. I had freedom to ease back into work at my own pace—started back after a month and still not back to full schedule. After returning to work my husband was unable to devote much emotional energy to grieving, so I have largely grieved without him (Cacciatore, 2008, pg 361)  [My] husband returned to work . . . and seemed happy to do so . . . and I was left on my own as his job took him out of town a lot . . .We have since divorced . . . He has moved on with his  career and has separated himself from his entire family . . . I work in the community and . . . feared returning to work and being overwhelmed (Cacciatore, 2008, pg 361) | Samuelsson et al, 2001; Cacciatore, 2008; Yamazaki, 2010; Avelin, 2013. |
|  | Return to social life |  | I didn’t go out socially for six [months]. My husband went to one get-together three [months] after our son’s death and came home within half an hour. I still cannot be around our friends’ daughter who was born on our son’s due date (Cacciatore, 2008, pg 361) | Samuelsson et al, 2001; Cacciatore, 2008; Yamazaki, 2010. |
| Need for Support | Support seen as a recognition of grief | Support and talking to people with similar experience viewed as validation of grief | . “other (grieving) parents who support me and have validated my grief. . . .. Once I got validation from others, things starting getting better for me.” (Cacciatore,2010, pg 139)  “I was so grateful to have a place to turn where people experienced the same nightmare and knew what I was going through...Others just can’t relate to what has happened.”. (Cacciatore and Bushfield, 2007, pg 67) | Cacciatore and Bushfield, 2007; Cacciatore, 2008; Cacciatore, 2010; Downe et al, 2013; Lee et al, 2013. |
|  | Support as comfort | The reversal of isolation and knowing that they are not alone in their grief | . “. . . having people acknowledge (her) life and death and her impact on my family, helped me to know that I’m not crazy.” (Cacciatore and Bushfield, 2007, pg 67) | Cacciatore and Bushfield, 2007; Cacciatore, 2008, Downe et al, 2013; Lee et al, 2013. |
|  | Professional support | Dos and Don’ts of health professionals | “There is a strong need to find a therapist that has experienced the loss . . . I’ve yet to find one that has experienced the death of a child . . . the most hurtful thing was never getting a call from our Drs. to see how we were doing.” (Cacciatore and Bushfield, 2007, pg 71)  “Don’t say, ‘You are young—you can have another’”(Kelly and Trinidad. 2012. Pg 144) | Samuelsson et al, 2001; Trulsson and Radestad, 2004; Cacciatore and Bushfield, 2007; Cacciatore, 2008; Kelly and Trinidad, 2012; Downe et al, 2013. |
| Recovery/Life changing event | Empathy | Reaching out to others who are grieving | .”. . . Although I’d give up all the growth in a second to have her back, her death has propelled me toward being more compassionate, helping others, and she has changed the way I view the world.” (Cacciatore and Bushfield, 2007, pg 68) | Cacciatore and Bushfield, 2007; Cacciatore, 2008. |
|  | Renewed sense of self | Change in the way they view themselves | “My child’s death has changed me to be a more sensitive person to other’s feelings. Her death has made me realize not to take things for granted such as my living children.” (Cacciatore and Bushfield, 2007, pg 68) | Cacciatore, 2008; Kelly and Trinidad, 2012; |
|  | Spirituality | Change in spirituality | Our child’s death] has made it harder for me to pray—I feel like God can’t or won’t take care of us now. (Cacciatore, 2008. Pg 360)  My husband is no longer an atheist . . . and believes that there is something after you pass (Cacciatore, 2008. Pg 360) | Cacciatore, 2008; Kelly and Trinidad, 2012; |
