# Supplementary material for: Exploring the intangible economic costs of stillbirth
Source: BMC Pregnancy Childbirth. 2015 Sep 1;15:188. doi: 10.1186/s12884-015-0617-x (PMC4556317; doi:10.1186/s12884-015-0617-x)
Supplement: Additional file 8: — Merging of themes. (DOC 29 kb) [file 12884_2015_617_MOESM8_ESM.doc]

## **Additional file 8:** Themes with similar ideas were merged and resulted in a total of four sections (A, B, C and D).

## **A: Depression/Profound grief**

The sub-themes for depression and profound grief were overlapping. For example, disappointment and shock (sub-themes in profound grief) are associated with depression while sub-themes from depression such as guilt and blame are closely linked to a sense of deep grief. This study found that these feelings were not transient and in some cases needed medical treatment. To estimate these psychosocial costs, the following questions are proposed:

- 1. Did you have any adverse psychological feelings after losing your baby?
  2. How soon after the stillbirth did these feelings start?
  3. How long did they last for?
  4. Did it affect your daily functioning?
  5. Did it affect your relationship with your partner, siblings or friends?
  6. Did you take time off work?
  7. Did you seek any professional support (medication, therapy, counselling) for these feelings?
  8. Was the support paid for privately or through the NHS?
  9. How long did the support last?
  10. Was the support helpful?

## **B: Need for support**

In many of the studies, participants spoke of the importance of support from family, friends and health workers. In particular support from health professionals resounded through all the studies. This was in terms of patients’ experiences of their interaction with healthcare staff (appendix 8) such as: The manner in which the news was broken to them; the adequacy and timeliness of the information; staff attitude to their feelings; advice on the next steps; and follow-up calls on the families to know how they were coping For this professional support to be effective, it was perceived that it should be a long-term project. The proposed questions therefore, are:

- 1. Was there any support from the health staff after you lost your baby?
  2. Was the help by a doctor, midwife or counsellor?
  3. In what form did the support come?
  4. How soon after the stillbirth did it start?
  5. How long after the stillbirth did it last?
  6. How often was it given?
  7. Was this support beneficial?
  8. Did you seek other forms of support?
  9. Was it privately or through the NHS?
  10. Was the support effective?

## **C: Social Isolation/return to normality**

These two themes were merged as it was felt that a return to normality especially in terms of work and social life could be hindered by the level of social isolation one experiences. To quantify these costs, the proposed questions are:

1. Were you able to freely talk about your loss to friends and colleagues?
2. Did you experience any form of stigmatisation following your loss?
3. Was your partner’s grief recognised by friends and colleagues?
4. How soon after the stillbirth did you/your partner go out socially?
5. How soon after the stillbirth did you/your partner go back to work?
6. On returning to work, would you say that you/your partner were working to your fullest productive capacity?

## **D: Couples’ relationship/siblings issues**

As both themes dealt with events in the aftermath of a stillbirth that occur within the family, it was only appropriate that the two themes were merged together. The proposed questions here are:

1. Did the stillbirth affect communication with your partner?
2. Did it affect your social activities with your partner?
3. Did it affect your sexual relationship?
4. For how long did these effects last?
5. Did it lead to a breakdown in the relationship?
6. Did you seek any professional help?
7. If you have other children, what effect did the stillbirth have on them?
8. If you have other children, did the stillbirth alter your relationship with them?
9. How long did these feelings or changes last?
10. If you have other children, did they require professional help?
11. If they required help, was it paid for privately or by the NHS?
